# American Medical Association

**Published:** 1888-06

**Authors:** 


### Proceedings of Sections.

### SECTION ON PRACTICAL MEDICINE, MATERIA MEDICA, AND PHYSIOLOGY.

C. W. Van Bibber, Baltimore, Chairman.

N. S. Davis, Jr., Chicago, Secretary.

The first subject considered was "Pneumonia: Etiology, Pathology, and Treatment," with a paper on "The Mechanism of Pneumonia and its Treatment," by C. W. Van Bibber, Baltimore.

It was discussed by Professors James T. Whittaker, Cincinnati, and N. S. Davis, Chicago.

This was followed by a paper on "Treatment of Empyæma," by Wm. C. Dabney, University of Virginia.

And one "On the Hippocratic Operation for Empyæma, with Illustrative Cases," by Frank Woodbury, Philadelphia.

Another was on "The Unity of Tuberculosis," by J. H. Musser, Philadelphia.

"My own Clinical Experience in the Administration of the Iodide of Potassium in the Treatment of Syphilis" was the subject of another paper by Morris H. Henry, New York.

"Diagnosis and Treatment of Diseases of the Stomach," was considered and a paper was read on "Some Practical Remarks on Diagnosis of Diseases of the Stomach," by Dr. W. Pepper, Philadelphia.

"Observations on Nutrition, with especial Reference to the origin of Fat, Urea, and Animal Heat," was the subject of a paper by Henry C. Chapman, Philadelphia.

"Hot air Bath in the Treatment of Rheumatism," by Chas. N. Sheppard, Brooklyn, was another paper read.

"Evolution of the Cystic Kidney," by I. N. Danforth, of Chicago, in which he said:

The causes of cystic or sacculated kidney may be classified as follows:

1. Diathetic causes: (a) excess of saline elements of the urine; (b) tuberculosis; (c) carcinoma.

2. Congenital causes: (a) floating kidney with consequent "twist" of ureter; (b) congenital hydronephrosis; (c) congenital degeneration of elements.

3. Mechanical obstruction consequent upon disease of the pelvic organs.

4. Traumatic causes.

5. Pathological cysts; dermoid cysts; hydatids; cystic metamorphosis.

By far the greater number of cystic or surgical kidney occur during the active period of life, when the peculiar diatheses which lead to them are most likely to manifest themselves, and this is peculiarly true of the first group of cases—those produced by an excess of the saline elements of the urine.

Tuberculous disease of the kidney does not often occur as a primary disease, but whether the deposit be primary or secondary, its behavior is substantially the same. The tubercular deposit is most common in the cortex, and generally each deposit clusters around some arterial twig.

In the adult, carcinoma of the kidney is rarely primary, and secondary manifestations of renal cancer are by no means common. Secondary cancer of the kidney is not generally followed by cystic degeneration. Nevertheless, this does occasionally happen, and in the following manner: Cancerous deposits in the kidney sometimes occur as infiltrations, sometimes as round nodular deposits. In the latter case each nodule is surrounded and isolated by a fibrous capsule, composed of hypertrophied connective tissue. The cancerous nodule is gradually shut off from its blood supply to an extent sufficient to induce early and rapid retrograde changes, fatty or mucoid degeneration, and the solid cancerous nodule becomes transformed into a soft pultaceous mass, or even into a dirty yellow or grayish fluid. This generally finds its way into the pelvis of the kidney and thence into the bladder. It sometimes happens that the first intimation of renal cancer comes from a profuse discharge of purulent or bloody matter per urethra.

Primary cancer of the kidney is a disease peculiar to children "before the fifth year." It is almost always of the encephaloid type, and may attain immense proportions. He had seen one case in which the tumor distended the abdomen to an almost incredible degree, while the alimentary canal, the liver, and other abdominal organs were compressed and crowded so as to be hardly recognized. In this case there were numerous cystic cavities, with fluid contents of a dirty gray color.

Cystic kidney produced by congenital causes: Migratory kidney with consequent twist or doubling of ureter.

General congenital cystic degeneration: At rare intervals an infant is born with an enormously distended abdomen, due to the fact that the kidney has been converted into a *congeries of cysts* by a process of general cystic degeneration. They are regarded as a form of cystic degeneration of the malpighian bodies, but occasionally the convoluted tubules appear to be involved.

Cystic disease of the kidney produced by mechanical obstruction consequent upon disease of the pelvic organs; diseases of the bladder which produce thickening of its wall.

He had recently seen a case of double hydronephrosis in a woman of about forty year of age, apparently caused by compression of the ureter by an enlarged and prolapsed uterus. The cervix uteri was hypertrophied, tilted up against the bladder, and fixed in this position by adhesions in such a manner that the ureters were constantly compressed.

Any pelvic disease which is followed by pathological new formations, or the dislocation of the pelvic organs, may produce compression of the ureters and cystic kidney.

The traumatic causes cannot be formally classified, but any injury of the kidney may result in cystic disease. He had seen a short time ago the case of a young man who fell down a rough and precipitous hillside, receiving some injury of the right kidney, which was followed by pain, tenderness, and hæmaturia. These symptoms shortly subsided and the patient regarded himself as quite well, although the kidney remained slightly tender. He had occasional, but not severe, attacks of nephralgia, and four years after the injury he died, after nephrotomy, the kidney being an enormous multiple abscess.

"On Headache from Overlooked Causes in the Naso-pharynx and Ears," by Henry Gradle, Chicago.

"Etiology of Typhoid Fever," by V. C. Vaughan, Ann Arbor, Michigan.

"Oxygen Enemata as a Remedy for Certain Diseases of the Liver and Intestinal Canal," by J. H. Kellogg, Battle Creek, Michigan.

### SECTION OF OBSTETRICS AND DISEASES OF WOMEN.

Ely Van de Warker, Syracuse, New York, Chairman.

E. W Cushing, Boston, Massachusetts, Secretary.

The meeting was opened by the President, Dr. Ely van de Warker, of Syracuse, New York, who delivered an address on the subject "How Gynæcology is Taught," in which he discussed the various methods of instruction, and concluded by paying a tribute to American gynæcology, and especially to its founder, Marion Sims.

Dr. Price, of Philadelphia, Pennsylvania, next followed on "Free Nursing as Organized in Philadelphia."

He described the system which had been there adopted of furnishing the indigent with skilled nurses, by which operators were enabled to perform even most difficult operations in the homes of the poor with proper assistance.

This institution was organized two years ago by two charitable ladies of Philadelphia. It is supported by voluntary contributions and such small amounts as the patients are able to contribute. The corps consists of seven well-trained nurses and their assistants. Two maternity nurses attend to the obstetrical part of the work, and separate nurses are employed for the care and attendance of contagious diseases.

The excellence of the plan is shown by the astonishing result of only one death in over ninety abdominal sections.

Professor W. W. Jaggard, of Chicago, Illinois, next followed with an essay entitled "The Relation of Endometritis Gravidarum to the Persistent Vomiting of Pregnancy," in which he included the report of a case of hyperemesis gravidarum caused by endometritis. The reader was of the opinion that even in the ordinary text-books the distinction was not sufficiently made between hyperemesis due to pregnancy and pregnancy incidental to the vomiting.

In the case reported, abortion was induced by introducing a bougie between the uterine walls and membranes after all other means for arresting vomiting had proven futile. Eight hours later the ovum came away and the placenta was removed according to Credé's method. The fœtus was of eight months' gestation. The abortion was immediately followed by cessation of the vomiting. That the vomiting was not due to an artificial displacement of the uterus was proven by the fact that it occurred as late as the sixteenth to the eighteenth week.

Professor W. H. Wathen, of Louisville, Kentucky, had seen but one case of truly uncontrollable vomiting. The patient was two or three months pregnant, and could retain absolutely nothing on her stomach. He at once dilated the cervix to the depth of one inch and a half by means of a steel dilator. Dilatation had previously been practiced by the attendant with sponge tents without affording relief. After the dilatation, the nausea and vomiting abated, but abortion occurred a few days later.

Dr. Daniel T. Nelson, of Chicago, narrated cases where vomiting was due to flexion, either of the uterus primarily, or due to the displacement of the ovaries or disease of the adnexa. He said in one case a complicating fibroid was the cause of flexion, and hence of the vomiting.

Drs. Maury, of Memphis, and Marcy, of Boston, participated in the discussion.

Professor Jaggard protested against dilatation of the cervix simply as a means of arresting vomiting, for in a large number of cases it terminates in the premature expulsion of the ovum.

Dr. William Goodell, of Philadelphia, next read an entertaining and humorous paper on the "Nervous Rectum." He described the rectum as being sometimes insane, as it were, whilst the rest of the body enjoys perfect sanity. One variety of this neurosis is the hysterical rectum, the muscles being thus affected, but the sphincters more so than the other set of muscles. A frequent form is spasm of the sphincter, which renders defecation painful, and hence induces costiveness. These victims become easily addicted to opium eating. When the rectum is loaded a pulsating pain is felt. If there be, in addition, some ovarian irritation and enlargement the affection becomes very distressing.

Another form the speaker alluded to as the "jealous" rectum. He gave some very ludicrous instances where the rectum put its veto on any attempts at indulging in social intercourse. One lady was in a perfectly comfortable condition until she put on her bonnet, when this unruly organ would begin to remonstrate audibly and continue to keep up in such a manner that the unfortunate possessor would be compelled to take off her bonnet and make up her mind to stay home. After this everything would again become quiet. Another lady always began to have an evacuation from the bowels as soon as she received a letter from her husband, and was obliged to delay reading it until the rectal demands had been satisfied. A third individual would soil her bed after any violent mental emotion. All these persons were kept prisoners at home and had to abandon social intercourse.

A third form he described as "follicular colitis" or "membranous enteritis." He found this affection so often in hysterical patients that he looked upon it as a neurosis, just as pruritus, shingles, etc., are nervous skin affections. He found all these forms of rectal trouble as being peculiar to emotional women of high intelligence; none of them belonged to the lower walks of life. The diagnosis between these neurotic troubles of the rectum and disease of the coccyx is readily made by the introduction of the index finger into the rectum and the thumb over the coccy, showing that this appendage is movable.

The treatment depends on the form of the trouble. He regarded that of Weir-Mitchell for nervous prostration as the best—seclusion, forced feeding, massage, and electricity, the latter two equalizing the nervous fluid and stimulating a healthy action of the nerves. As soon as enemata can be borne, they should be administered before bedtime. Suppositories of iodoform act beneficially, and, in case of spasm of the sphincter, stretching of the sphincter ani. Follicular colitis is almost incurable, but may be soothed by means of suppositories of iodoform, antipyrin, etc. Sometimes injections of lime water or carron oil greatly relieve this trouble. He cautioned strictly against the use of opium in any form, as these patients are very apt to become opium eaters. Altogether the best medication consists in the administration of remedies constitutional in their action. His favorite prescription is the pil. sumbul comp.; Bland's pill is an excellent remedy, beginning with one pill three times a day, gradually increased until three are taken after each meal. Occasionally he gave as many as five after each meal. Pills composed of the three valerianates (zinc, iron, and quinine) are of great value, also pills composed of chloride of gold and soda. When malaria is at the bottom of the trouble, Fowler's solution acts well. The bromides are often needed and may be advantageously combined with the bitter tonics, as the tinct. of gentian comp. When the paroxysms are sudden, antipyrin and the hydro-bromate of hyoscine are serviceable. Absolute rest of mind and body secured by absolute seclusion in a darkened room is sometimes indispensable.

A paper was read by Dr. L. H. Dunning, of South Bend, Indiana, on "Double Uterus and Vagina," with the following conclusions:

1. Congenital malformations of the uterus or vagina are more frequent than is usually supposed.

2. Of the different anomalies, uterus bicornis is the most frequent form—over 50 per cent.

3. The fecundity of women thus affected is not materially diminished.

4. As a result, however, difficult labors are more numerous.

5. In all established cases of uterus bicornis pregnancy has thus far occurred.

6. The forms of malformation of the uterus, in which abortion or miscarriage are most apt to occur, are the *uterus didelphys* and *uterus bilocularis*.

7. Both divisions may be pregnant at the same time and in different stages.

8. Disorders of menstruation are very apt to occur.

9. The amount of the menstrual fluid, when normal, is small.

10. Menstruation may occur simultaneously from both uteri, or one may alternate with the other.

11. The disturbances caused by these anomalies show the important part that the uterus plays during female life.

"Abdominal Section in Extra-Uterine Pregnancy," by W. H. Wathen, Louisville, Kentucky.

"The Value of Galvanism as applied by Apostoli in the Treatment of Fibroid Tumors of the Uterus, with Cases," by Franklin H. Martin, Chicago.

Dr. A. B. Carpenter, of Cleveland, Ohio, next described "A New Method for Supplying the Electrolytic Current in Uterine Fibroids." The author spoke of the difficulty of keeping the ordinary physicians' batteries in good running order, and therefore devised a method of supplying the electric current at any time desired in the proper strength. He took his current directly from the street, using the electric current as furnished by the Edison light for this purpose. The Brush light is too dangerous, but the Edison system, which furnished an electric intensity of 100–110 bolts, will not prove dangerous, even with the entire strength outside of the rheostat. The author exhibited the working of his apparatus, with the wires attached from the street.

The following papers were presented:

"The Infant Food Problem," by Professor W. B. Atkinson, Philadelphia.

"Puerperal Hæmorrhage," by Mary H. Thompson, Chicago.

"Report of Forty-seven Cases of Alexander's Operation; Results of Observations and Simple Mode of Operating," by J. H. Kellogg, Battle Creek, Michigan.

SECTION ON SURGERY AND ANATOMY.

Donald McLean, Detroit, Michigan, Chairman.

B. A. Watson, Jersey City, New Jersey, Secretary.

Address by the Chairman, "Some Thoughts on Retrospective and Prospective Surgery," Donald McLean, Detroit, Michigan.

"The Appendix Vermiformis; its Functions, Pathological Changes, and Treatment," by Henry Hollingsworth Smith, Philadelphia.

"The Cæcum and Appendix; their Relations in Health and Disease," by Joseph Ransohoff, Cincinnati.

"A Case of Typhilitis with Perforation; Laparotomy; Recovery," by L. S. McMurtry, Danville, Kentucky.

The subject "Intestinal Obstruction in its Surgical Aspects," was discussed and a paper was read.

"Rectal Insufflation with Hydrogen Gas as an Infallible Diagnostic Measure in ascertaining the Existence of Visceral Injury of the Gastro-Intestinal Canal in Penetrating Wounds of the Abdomen, illustrated by Three Experiments," by N. Senn, Milwaukee, Wisconsin.

The paper, a voluminous one, comprises sixty-four experiments, with descriptive remarks and comments. After making experiments on both animals and the human subject, and carefully studying the details of each, Professor Senn expresses himself in the following manner: After a careful study of the subject of rectal insufflation of hydrogen gas in its various aspects, I do not hesitate to recommend its adoption in practice as an infallible diagnostic test in demonstrating the existence of a wound of the gastro-intestinal canal in penetrating wounds of the abdomen, without resorting to an exploratory laparotomy. In conclusion I beg leave to submit for your discussion the following propositions:

1. The entire alimentary canal is permeable to rectal insufflation of air or gas.

2. Inflation of the entire alimentary canal from above downwards through a stomach-tube seldom succeeds, and should therefore only be resorted to in demonstrating the presence of a perforation or wound of the stomach, and for locating other lesions in the organ or its immediate vicinity.

3. The ileo-cæcal valve is rendered incompetent and permeable by rectal insufflation of air or gas under a pressure varying from one-fourth of a pound to two pounds.

4. Air or gas can be forced through the whole alimentary canal from anus to mouth under a pressure varying from one-third of a pound to two pounds and a half.

5. Rectal insufflation of air or gas, to be both safe and effective, must be done very slowly and continually.

6. The safest and most effective rectal insufflator is a rubber balloon large enough to hold sixteen litres of air or gas.

7. Hydrogen gas should be preferred to atmospheric air or other gases for purposes of inflation in all cases where this procedure is indicated.

8. The resisting power of the intestinal wall is nearly the same throughout the entire length of the canal, and in a normal condition yields to diastaltic force of from eight to twelve pounds of pressure. When rupture takes place it either occurs as a longitudinal laceration of the peritoneum on the convex surface of the bowel, or as multiple ruptures from within outwards at the mesenteric attachment. The former result follows rapid and the latter slow inflation.

9. Hydrogen gas is devoid of toxic properties, non-irritating when brought in contact with living tissues, and is rapidly absorbed from the connective tissue space and all of the large serous cavities.

10. The escape of air or gas through the ileo-cæcal valve from below upwards is always attended by a blowing or gurgling sound, heard most distinctly over the ileocæcal region, and by a sudden diminution of pressure.

11. The incompetency of the ileo-cæcal valve is caused by a lateral and longitudinal distension of the cæcum which mechanically separates the margins of the valve.

12. In gunshot or punctured wounds of the gastro-intestinal canal, insufflation of hydrogen gas enables the surgeon to demonstrate positively the existence of the visceral injury without incurring the risks and medico-legal responsibilities incident to an exploratory laparotomy.

"The Surgical Advantages of the Buried Animal Suture and its Adaptability to Special Purposes," was the title of a paper by Henry O. Marcy, Boston.

A paper was also read on "Sarcoma of the Scalp," by Professor P. S. Conner, Cincinnati.

"On the Radical Cure of Varicocele Attended with Redundancy of Scrotum," by Morris H. Henry, New York.

"A New Method of Controlling Hæmorrhage," by C. S. Muscroft, Cincinnati.

"Causes Determining the Localization of Infection; an Experimental Study," by Bayard Holmes, Chicago.

SECTION ON STATE MEDICINE.

H. B. Baker, Lansing, Michigan, Chairman.

S. T. Armstrong, Marine Hospital Service, Secretary.

Address by the Chairman, H. B. Baker.

"Should the National Government Defend our Ports Against the Invasion of the National Enemy, Contagious Diseases?" by Benjamin Lee, Philadelphia, Pennsylvania.

"Uniform Medical Practice, Report by a Committee of the Association," by Perry H. Millard, Chairman.

"Cremation of Garbage," by J. Berrien Lindsley, Nashville, Tennessee.

"House Drainage," by Joseph F. Edwards, Philadelphia, Pennsylvania.

"Personal Prophylaxis Against Malarial Diseases," by Geo. H. Rohe, Baltimore, Maryland.

"Atmospheric Temperature and Intermittent Fever," by Henry B. Baker, Lansing, Michigan.

"Abstract of Laveran's Researches on the Hæmatozoön of Malaria," by S. T. Armstrong, U. S. Marine Hospital Service.

"What Evidence is there for a Malarial Germ," by Samuel N. Nelson, Boston, Massachusetts.

"The School-room a Factor in the Production of Disease," by J. A. Larrabee, Louisville, Kentucky.

"Recent Discoveries and Researches with Regard to a Common Cause of certain Infectious Diseases," by C. W. Chancellor, Baltimore, Maryland.

"The Causation of the Autogenous Fevers," by Victor C. Vaughan, Ann Arbor, Michigan.

"Eastern Carolina as a Residence for Tuberculous Patients," by J. M. Baker, Tarboro, North Carolina.

SECTION ON OPHTHALMOLGY, OTOLOGY, AND LARYNGOLOGY.

F. C. Hotz, Chicago, Illinois, Chairman

John L. Thompson, Indianapolis, Indiana, Secretary.

Address by the Chairman, F. C. Hotz.

"Melanotic Disease of the Conjunctiva and Episcleral Tissue, with Five Illustrations," by Robert Sattler, of Cincinnati.

"The Treatment of Strabismus due to Paralysis or Extreme Over-correction, with Loss of Motion," by A. E. Prince Jacksonville, Illinois.

"Some Practical Suggestions Regarding Certain Anomalies of the Ocular Muscles," by George T. Stevens, of New York.

"Binocular Astigmatism," by H. Culbertson, of Zanesville, Ohio.

"The $\frac{1}{4}$ Dioptric Cylinder Lens the Most Useful of the List of Cylinders," by J. J. Chisolm, of Baltimore.

"The Different Methods of Adjusting Lenses," by Dudley S. Reynolds, of Louisville.

"The Advantages of Leaving One Eye Open during the After-treatment of Cataract Cases," by J. J. Chisolm, of Baltimore.

Two years before, the subject of light dressings was brought before the section, when Professor Chisolm stated that he would test the proposed method and report the result of his experiments at the next meeting of the Association. Last year at the Chicago meeting he reported to the section on ophthalmology that for twelve months he had abandoned compresses, bandages, dark rooms, and restraint in the after-treatment of his cataract-extraction cases, and that he was both surprised and delighted at the results obtained. The cases as a rule behaved so much better under the adhesive-strip dressing, the eyes were so much less watery, less injected, so much less flinching when exposed to the strong light of day than when dressed by compresses and bandages and kept in bed in dark rooms, that he was forced to recognize the improvement in contrast, and was forced to accept the conclusions that much of the irritation found in eyes when the restraining treatment was used was the direct result of the bandaging and not of the operation.

In his report of cataract-work at the Presbyterian Eye and Ear Charity Hospital of Baltimore City, at which hospital he controls a large amount of clinical material, he gives the result of his further experience in the after-treatment of cataract cases. During the past year in seventy-four cases of simple senile cataract he closed only the eye operated upon, and that with a piece of isinglass plaster one and one-half by one inch in size. When properly adjusted and dried the lids are closed together as if one piece; the canthi are not covered, so that from them the eye-secretions can escape; and eye drops may also be instilled, should they be needed, without disturbing the adhesive plaster dressing. If properly put on this piece of isinglass plaster will remain till removed on the fourth or fifth day. The other eye is left open for the guidance of the patient.

The patient is never operated upon in his bed, but always upon a narrow table of suitable height, placed before a large window. During all the steps of the operation a septic and antiseptic treatment is used. Solutions of the biniodide of mercury, 1 to 20,000, is the germicide employed. When the operation is smoothly done he believes that nine-tenths of the dangers have already been obviated. When the plaster on the newly operated eye is dry, the patient gets down from the operating table and walks to his room, where he may lie down or move about at his pleasure. Having one eye open he can see to eat, dress himself, and converse with friends. No restraints are imposed beyond not using the eye for reading, and keeping in a moderately lighted room and out of the sunlight. The strip of bandage is removed on the fourth or fifth day. One eye having been always open, the covered one has never become sensitive to the light. When it is finally uncovered no smoked glasses are needed, and the eye shows no irritation from the exposure. He says in his paper that it matters not how beautiful and acceptable the theory that absolute quiet and rest under restraint must prevent friction and irritation, and consequent inflammation, yet experience will show that exposure does not bring about the train of irritable phenomena which our long-established theory would predict.

In his year's work of 74 simple senile cataract extractions, all of them treated by the closure of one eye only, there had not been the loss of a single eye. In only two was the pupil closed, necessitating in the future a secondary operation for the restoration of sight; and that such a result has and will continue to take place from time to time in the hands of the most careful surgeon who practices all the restraints of bandages and dark rooms. For 25 years he bandaged and confined every cataract patient. He says that for the past two years he has abandoned bandages and restraint, and that he has had better results under the new method of dressing than he ever had before, and that now he has not the courage nor the cruelty to impose the needless and, as he says he now knows it to be, injurious bandages.

He knows that the plan of simple dressings and after-treatment is a revolution. The plan has in individual instances been forced upon the notice of every ophthalmic surgeon by the obstinacy or unruliness of obstreperous patients. Professor Chisolm has been convinced by such incidents that a system of simple after-treatment is best, and must eventually be adopted by ophthalmic surgeons.

In closing he says that if each surgeon would, in alternate cases, use the bandage and restraint with the one, the light dressing and no restraint with the other, and in every other respect by careful operation and antiseptic treatment start the patients alike up to the after-dressing, he would find that all the patients would do so equally well that they would be forced to diminish the annoyances which they have heretofore thought it their duty to impose.

"Some Observation on the Extraction without an Iridectomy and use of the Bandage in the After-treatment," by Geo. E. Frothingham, of Ann Arbor.

"Gummata of the Ciliary Region," by S. C. Ayres, of Cincinnati.

"Some Remarks on Diseases of the Labyrinth," by Franc Dowling, of Cincinnati.

"The Bougie in Catarrhal Inflammations of the Middle Ear," by W. Cheatham, of Louisville.

"Passive Motion in the Diagnosis and Treatment of Middle-Ear Affections, with a New Otoscope (pneumatic)," by S. S. Bishop, of Chicago.

"Ocular Troubles Influenced by Nasal Diseases," by L. H. Taylor, of Wilkesbarre, Pennsylvania.

"Reflex Nasal Cough, with Report of Cases," by Max Thorner, of Cincinnati, Ohio.

"Reflex Phenomena in Childhood caused by Rhinitis," by J. A. Stucky, of Lexington.

"Naso-Pharyngeal Fibromata," by E. F. Ingals, of Chicago.

In 1884 he presented to the same section a paper on naso-pharyngeal fibromata in which he reported four cases, two of which had been cured, and two of which had disappeared, so that the result was unknown. In that paper he advocated the extirpation of these growths, when possible, through the natural passages, by means of the galvano-cautery ecraseur,or other methods employed by laryngologists in the destruction of nasal polypi in preference to the formidable operation including removal of the superior maxilla. Dr. Lincoln, of New York, has shown that the operation usually adopted by surgeons gives a mortality of over 25 per centum, while it is not very successful in preventing recurrence, as in only about 14 per centum is it certain that the tumor will not return within a year. By the method which Professor Ingals then recommended, it was shown that the danger to life was very much less, and that the ultimate results were much better. In over 50 per centum recurrence had not taken place within twelve months. Recently, one of the cases then reported which had failed to return after the first sitting for completion of the operation, had returned after the lapse of five years with a renewed growth in the same locality. This he removed by means of the galvano-cautery ecraseur. The operation was done after the parts had been anæsthetized by cocaine, and was completely successful in removing all the tumor, which consisted of three lobules attached by a base about half an inch in diameter at the vault of the pharynx and posterior naris of the left side. The tumor removed five years ago had the appearance of being composed entirely of fibrous tissue. Of the tumor recently removed, the smaller lobules, which measured about five-eighths of an inch in diameter, proved upon microscopical examination to be fibro-cellular in character, about one-half fibrous; and the largest, which measured about one by two inches in diameter, seemed also fibro-cellular, about three-quarters fibrous. He also presented the history of another somewhat similar case in which a tumor in the nasal cavity was removed by the combined means of electrolysis and the galvano-cautery snare. Details were very fully given, for which space cannot be found in this abstract.

Dr. Robert Tilley, of Chicago, Illinois, reported a case of congenital atrophy of the cilia and supercilia, associated with atrophy of all the nails of both fingers and toes. The subject of the report was a girl of seventeen years. The cilia, especially of the lower lids, were absent, and the supercilia consisted of short, stubby hairs averaging about one-eighth of an inch in length, and presenting the appearance of having been burnt off. There was not a single nail on either fingers or toes but exhibited similar signs of imperfect development. They gave no signs of change of color, nor had they lost any of the usual lustre of a normal nail. They were all remarkably thin, and each nail exhibited from one to three depressions on the anterior part of the nail. There was no projecting free border to any of the nails. The cilia and supercilia, both in the shaft and root, were atrophied, and some of the longer ones clearly exhibited partial breaks in the shaft, which probably accounted for the burnt-off appearance. The girl claimed that the cilia and supercilia, as well as the nails of her father and two brothers, were just like hers. This was found by Dr. Tilley to be true relative to a younger brother, but the father and the older brother could not be seen.

"Tubercular Laryngitis," by D. E. Welsh, of Grand Rapids, Michigan.

"The Tonsils as a Cause of Naso-Pharyngeal and Aural Disorders," by H. Gradle, of Chicago.

"A Case of Atrophy of the Supercilia and Cilia Associated with Atrophy of all the Finger Nails," by Robert Tilley, of Chicago.

"Tobacco Amblyopia," by A. R. Baker, of Cleveland, Ohio.

"Fatty Tumors of the Orbit," by Jas. H. Buckner, of Cincinnati.

### SECTION ON DISEASES OF CHILDREN.

Professor F. E. Waxham, Chicago, Illinois, Chairman.

Dr. W. B. Lawrence, Batesville, Arkansas, Secretary.

"Infant Feeding: I, Foods for Prematurely Born Children; II, Mixed Diet; III, Artificial Foods; IV, Feeding in Acute Disease," by C. W. Earle, Chicago, Illinois.

He presented, by way of introduction, a résumé of Baginsky's recent work on Diseases of Children, and said:

The greatest mortality in all climes and among all nations is due to the lack of mother's milk. In Berlin, one-half of all children born out of wedlock die within six months, and during the summer months a very great number of all children succumb to errors of diet.

Hence the most important question is infant diet. A division may be made of infant foods into two groups.

Next to mother's milk comes the wet-nurse.

In the first group are enumerated:

Cow's milk, peptogenized milk, condensed milk, cream mixtures, and Liebig's foods.

In group two are found the prepared foods.

The difference between mother's milk and cow's milk is given in general terms as follows: The water is about equal, casein, albumen, salts, and butter a greater, and of sugar a smaller, quantity in cow's milk.

This analysis as regards the fats does not agree with the usual analysis. He also says that coagulation and fermentation arise sooner in cow's than in mother's milk. Out of cow's milk it is impossible to manufacture a substitute which in every essential is equivalent to mother's milk. The greatest difficulty is to obtain good cow's milk, and this is why condensed milk is frequently the best substitute which can be provided. Cow's milk if given to babies should not be administered raw, but boiled long and thoroughly to destroy bacteria. Germs of tuberculosis can be effectually destroyed if they exist in cow's milk by thorough boiling. Soxhlet's cooking apparatus is spoken of as the best and safest to use for the purpose of destroying the germs found in milk. By the use of this process cow's milk is much better as a child's food than we have formerly thought. Cow's milk should usually be prepared in proportion of one to three for young children, later in equal parts. Some children at nine months can take clear milk and digest it perfectly. A curious fact developed in the dilution of cow's milk, is that from at first being one to three this proportion is lessened about 15 per cent. during the first two months, and then increased until equal parts of water and cow's milk are used. Two methods of preserving milk are presented. By the first it is condensed with, and by the second without, sugar. The great difficulty in the condensing process is that too much sugar makes it indigestible, and yet if a milk of this kind is diluted so that it can be digested it is then too weak. Baginsky does not regard peptonized foods with great favor. Biedert's cream mixture contains only one per cent. of casein, and agrees excellently well in a large number of cases. Liebig's foods and soups are made from milk, wheat, and malt, but it is believed that the cream mixture mentioned above, when fresh and properly made, is better than the Liebig food. Nestlé's food is spoken of as being the best type of a prepared food. It contains forty per cent. of sugar, five of fat, fifteen of proteid compounds and thirty per cent. of dexterine and starch. It has been noticed that a long continued administration of an artificial food, in some cases at least, brings about a change of blood. The red corpuscles diminish, but can be increased if a wet-nurse or mother's milk is substituted.

Professor Earle said that he agrees with the old author, who said that "Nature does not afford, nor can art supply, a substitute for mother's milk," and he thought that medical men should always try to induce mothers to nurse their babies in suitable cases.

We should explain the dangers which children pass through, particularly during the summer, and state plainly the probabilities that a certain number of them cannot survive upon any artificial food.

His belief that no artificial food can be selected which will furnish a proper nutriment through the hot months to a considerable number of these little people is so firm that he is every year, with greater frequency, suggesting a wet-nurse. A mixed diet is preferable to one wholly artificial—that is, part mother's or nurse's milk, with the remainder supplied from some outside source. One of the first conditions where an artificial food for a baby is demanded is when the child is prematurely born. In some of these cases, the milk secretion of the mother is at once established. But in others several weeks may elapse before the milk secretion is established. The author has had charge of three infants born at six and a half months, in which artificial food had to be provided, and which he succeeded in saving by the use of artificial food. Cream is the basis, barley water the menstrum, to which is added a little salt, a little sugar of milk, and a small amount of lime water.

A prematurely born baby should be fed on a small quantity frequently. Sometimes not more than a half teaspoonful, and when not fed from the spoon, let an ordinary ounce bottle be provided with a rubber mouthpiece. For the first few days, perhaps, cracker water, with a small amount of sugar of milk is all that is necessary. Then cream added to a little cracker water, or cream and rice water; then cream and barley water, and, if vomiting takes place, a small amount of lime water should be added. If from any reason the cream does not agree with the baby, condensed milk with barley water, a little salt, and possibly the addition of a little lime water. By varying the amount of cream, using either barley water or rice water as the diluent, adding a little sugar of milk and a few grains of salt, and sometimes a little condensed milk, if the cream disagrees with the infant, one is able to carry along these puny children until such time as the milk secretion is established.

Speaking of the fermentation of milk, he said: Fermentation of milk occurs only in consequence of the introduction into it of micro-organisms. If the milk be received by a sterilized tube into a sterilized receptacle directly from the udder of the cow, it will not ferment, nor become acid, though kept indefinitely. It can be sterilized by heating to 70° for an hour, but in order to to kill the spores it is necessary to repeat the process for an hour each day for four or five days. Heating to 100° by a current of steam for one hour will sterilize it completely, but boiling coagulates the albumen and to some extent changes the milk sugar. The first process in the fermentation of milk is due to the action of a bacillus, and consists in the conversion of the milk sugar into lactic acid. This process ceases after a small quantity of acid is formed; but if the acid be neutralized by chalk, the fermentation will go on until the milk sugar is all decomposed. By the change of reaction of the milk the casein is coagulated. This coagulation is said to be due to the action of the acid and not directly to that of the bacillus. When the milk sugar is converted into lactic acid, another bacillus —bacillus subtilis—attacks the lactic acid and converts it into butyric acid, with evolution of carbon dioxide and free hydrogen. This bacillus cannot act on milk sugar unless it is first converted into lactic acid.

Under exceptional circumstances there is formed in milk a substance first discovered by Professor Vaughan, of Ann Arbor, and named by him "tyrotoxicon" (cheese poison). This is a crystalline nitrogenous substance, and is supposed to be a ptomaine. When taken, it produces pain at the base of the brain, vomiting, and purging. Professor Vaughan believes this to be the cause of cholera infantum. Tyrotoxicon is formed spontaneously in milk after some months; but it will be produced very quickly if some milk in which it has been formed be added to fresh milk. Its formation seems to be connected with the butyric acid fermentation.

Dr. I. N. Love, of St. Louis, Missouri, read a paper entitled "Membranous Croup and Diphtheria; are they Identical?" in which he maintained the position that membranous croup and diphtheria are identical.

He said that whether the membrane, as in the so-called true croup, be a fibrinous exudation, superficial and easily stripped from the surface, leaving a smooth mucous surface only robbed of its epithelium, while that of diphtheria is more of a coagulation penetrating or poured into the mucous tissues, a necrosis as it were, in which the eschar can be removed only with great difficulty, is not important, it being largely dependent upon the anatomical characteristics of the parts involved. That the disease is due to a special germ or micro-organism is admitted, and the recognition of this pathological point in the treatment has made a much more favorable showing in the mortality reports. One fact which is worthy of notice, and which is an additional argument in favor of the identity of the two diseases, is that the classical treatment for croup has for years been free exhibition of the mild chloride of mercury, coupled with stimulation, with a view to its defibrinating effects. The secretory system has thus been stimulated, and the effect has been to favor the moistening and exfoliation of the exudation and to antagonize the disposition to constitutional involvement.

Since the same plan of treatment has been applied to general diphtheria the mortality has diminished. He claimed that by the prompt recognition of the first appearance of diphtheria, and the immediate institution of active interference in the way of free purgation with the mild chloride of mercury, local antiseptics, and the continuance of constitutional measures, germicides and stimulators of glandular action, the bichloride of mercury, benzoate of sodium and large quantities of water, we can without doubt claim accomplishments that are tangible and positive.

"The Surgical Treatment of Empyæma," by D. A. K. Steele, Chicago, Illinois.

More than one-half of the cases of empyæma recorded occur during the first decade of life, and more errors of diagnosis are made in this than in probably any other thoracic affection.

It was his belief that a purulent pleuritis is due to auto-infection of a more or less limited area of the inflamed or damaged pleura through the medium of the general circulation. Other methods of infection are the use of unclean aspirating needles, rupture of a bronchus, extension of a septic pneumonia, secondary infection from the primary foci in some other point of the body, tubercular infection, etc.

The exact nature of fluid in the pleural cavity can only be ascertained by the introduction of a hypodermic needle. If, following the usual symptoms of an acute pleuritis, we have chills, fever, hectic, diarrhœa, and sweating, we are reasonably certain of our diagnosis without the aspirating needle.

All cases of pneumonia that are tardy and undergoing resolution should awaken a suspicion of empyæma. Secondary infection of the pericardium is a fatal termination in a number of cases and runs a rapid course. Empyæma, if left to itself, may terminate in recovery in one of three ways, according to L. Emmet Holt: (1) By spontaneous absorption of the purulent effusion; (2) by evacuation through a bronchus; (3) by opening externally through the intercostal muscles. But when left to nature the mortality is appalling. Rilliet and Barbex mention thirty-three cases, with four recoveries, twenty-one deaths, and eight not accounted for.

*Surgical Treatment.*—No absolute rule can be laid down for the management of all cases. We may resort, *first*, to aspiration; *second*, aspiration and washing out of the cavity with an antiseptic solution; *third*, thoracentesis with trocar and cannula; *fourth*, thoracentesis with subaqueous drainage; *fifth*, simple incision; *sixth*, simple incision and drainage; *seventh*, simple incision with through-and-through drainage, with or without antiseptic precautions; *eighth*, subperiosteal resection of rib, and drainage; *ninth*, thoracoplasty (Eslander's der's operation); *tenth*, perflation.

As practical surgeons we may confine ourselves to the relative merits of but two of these methods—aspiration and free incision with drainage.

In a minority of cases in children, simple aspiration, once or twice repeated, will effect a permanent and satisfactory cure.

The records of four such cases were presented.

Aspiration has the advantage of being simple, safe, and occasionally curative. But, if we find the fluid rapidly accumulates, or septic symptoms are developed, free incision with drainage is imperatively demanded.

Aspiration has the disadvantage of leaving a small amount of pus for reabsorption, or which may become an inspissated residuum that will result in a secondary abscess in after life. Surgically considered, it is not as perfect an operation as evacuation by a free incision and the introduction of a large size drainage-tube.

Of 121 cases treated by aspiration alone, twenty-three, or 19 per cent., were cured, six died, and the rest came to some other method of treatment, usually incision.

*Incision.*—Free incision therefore remains as the method possessing the decided advantage of complete evacuation of the purulent fluid, flakes of lymph, etc., and it moreover permits an exploration of the cavity with the finger or sound, and the breaking down of any septa or bands of adhesion. It permits of subsequent irrigation, if the septic nature of the fluid demands a washing out, and also gives complete drainage.

The technique of the operation is comparatively simple, the diagnosis having been established beyond a doubt by preliminary hypodermic aspiration. The field of operation is prepared in the usual manner for all aseptic operations. The point of selection for free incision and drainage is the posterior border of the axillary line in the sixth or seventh interspace. General anæsthesia is usually not required, although a few whiffs of chloroform in nervous children are of advantage sometimes. But local anæsthesia by means of the ether spray or hypodermic injections of from ten to fifteen drops of a 4 per centum solution of muriate cocaine answers every purpose in alleviating pain. The fold of skin at the site indicated is transfixed on a line with the upper border of the rib and incised for one and a half inches; then the intercostal muscles and pleura are divided, and as pus flows out in a full stream a large sized rubber drainage-tube is inserted and secured by safety pin, or by a button of rubber, flange reflected so as to prevent its intrusion into the pleural cavity. The tube should not be more than three inches long. Irrigation should not be resorted to except in septic empyæma where the fluid is fetid and caseous.

As soon as the tube is introduced it should be covered with sublimated gauze, and the pus should be allowed to flow out upon absorbent cotton or antiseptic gauze, and the dressings changed daily. Strict antiseptic precautions are always necessary, and our greatly improved results and lessened mortality are due to the antiseptic treatment now employed.

Dupuytren operated in fifty cases, with only four recoveries, while L. Emmet Holt reports sixty-three cases operated upon under full antiseptic precautions, with only two deaths. The average duration of the discharge was about six weeks. In children we are usually able to discard the use of tubes in three weeks. When, from the large size of the pleura, effusion occurs in close apposition to the ribs, flattening of the tube during pressure from coughing, or neuralgia due to pressure upon the intercostal nerve, subperiosteal resection of one and one-half inches of the single rib will afford ready relief and better drainage, although it is rarely required in old empyæmas in children. When we have a chronic abscess, discharging through a sinus and shortening the life of the patient by lardaceous degeneration of kidneys, liver, or spleen, or from general exhaustion, Estlander's operation of thorocoplasty should always be performed, although its field of usefulness is limited; but, if necessary, a sufficient number of ribs and a lung section of each should be made to permit the complete retraction of the divided ends of the ribs to the bottom of the cavity, thus closing it; otherwise a fistulous tract remains requiring a subsequent operation.

"Hepatic Incompetence in Children," by M. P. Hatfield, Chicago, Illinois.

"Chorea," by Geo. Wheeler Jones, Danville, Illinois.

"Infant Food, or the Care of the Infant," by J. M. Dunham, Columbus, Ohio.

"Tracheotomy in Pseudo-Membranous Laryngitis," by C. G. Jennings, Detroit, Michigan.

"Report on Intubation in Pseudo-Membranous Larnygitis," by F. E. Waxham, Chicago, Illinois.

"Antifebrin as an Antipyretic in the Treatment of Febrile Diseases of Children, with a Report of Fifty Cases," by F. J. Parkhurst, Danvers, Illinois.

SECTION ON MEDICAL JURISPRUDENCE.

E. M. Reid, Baltimore, Maryland Chairman.

C. B. Belt, Boston, Massachusetts, Secretary.

Address by the Chairman of the Section, E. M. Reid, Baltimore, Maryland.

"Paralytic States, their Relation to Testamentary Capacity," by E. C. Spitzka, New York.

"Medical Jurisprudence in Relation to Wills," by Richard Gundry, Baltimore, Maryland.

"Fœticide, its Increase, and Inadequacy of Law for its Prevention and Punishment," by H. C. Markham, Independence, Iowa.

"Expert Testimony in Medical Jurisprudence," by Orpheus Everts, College Hill, Ohio.

"Some Points in the Medical Jurisprudence of Insanity," by E. N. Brush, Philadelphia, Pennsylvania.

"Jurisprudence of Insanity," by T. Wright, of Bellefontaine.

"Insanity; Some Points of Medico-Legal Interest," by Philip Zenner, Cincinnati.

"The Influence of Experimental Science upon Law," by Clark Gapen, Chicago, Illinois.

"The Relation of Meconeuropathia, Opium Toxæmia and the Opium Psycho-Neurosis to Law," by C. H. Hughes, St. Louis, Missouri.

Professor Daniel R. Brower, of Chicago, read a paper on "The Medico-Legal Relations of Hysteria." The relations of hysteria to the law are often very perplexing, as they are not infrequent. The close relation that this disease bears to insanity makes the question of the legal responsibility of its victims often exceedingly doubtful. In order to properly estimate the legal aspects of the subject we must have a due conception of the disease. He takes exception to all opinions of its pathology that grow out of the misleading idea that originated the name.

Hysteria is a *psycosis;* the primary derangement is in the higher cerebral centres. The lower centres of the brain, the spinal cord, and symphathic nervous system are only secondarily affected. It consists essentially of a depressed condition of the intellectual and volitional areas of mental action, and an excitation of the emotional and perceptional areas. This condition of mind produces a defective power of the will; an imperfect self-control; a life largely governed by irresistible impulses; an isatiable craving for sympathy; an exceedingly irritable and irascible disposition; and with this is very often associated, sometimes as an inheritance, sometimes as an outgrowth of this unbridled emotional condition, a defective moral tone, and sometimes an unnatural and ungovernable activity of the sexual system. These people are deceitful, perverse, and obstinate; they practice, or attempt to practice, the most aimless and unnatural impositions. In illustration of the relation of hysteria to the crime of murder, two cases that have recently been before the courts in Chicago were cited.

The first case, of Adelina Roberts, a French woman, aged 42, who has a history of hysterical convulsions, aphonia, paralysis of an arm, paralysis of a leg, and hemi-anæsthesia at various times and of degrees of severity for a period of about twenty years. During all this time she was carrying on an illegitimate *liaison* with a Chicago merchant. He finally grew tired of her demands for money and attention, and tried to rid himself of her, but she would not be cast off. He stopped her allowance of money, and positively refused to have any further intercourse with her. This was followed by many days of convulsions, during which she was under my treatment at St. Joseph's Hospital in this city (Chicago). On her recovery from these convulsive seizures, and after her discharge from the hospital, during another exciting interview with him in his office in the presence of a number of employés, she shot him. After a long trial the jury declared her insane and sent her to the Hospital for the Insane at Elgin, Illinois. There she spent about three years, and was finally discharged under a writ of *habeas corpus.*

The second case was that Theresa Sturlatta, an Italian woman, aged 26, who had a history of hysterical convulsions, hemi-anæsthesia, catalepsy, the trance states, during a period of ten years. She killed her lover in his room at the Palmer House. The night before the killing she rode out with him to a place of public resort on the outskirts of Chicago, where they had a private room together, and he left her promising to return. It was a cold night in November. She waited for him until a late hour, when, upon making inquiry, she found that he had driven off without her. She had no money, and was therefore compelled to walk into the city, a distance of about five or six miles. It was raining at the time and she was menstruating. That night, on her return to Chicago, she was in a state of most intense hysterical excitement, and her menses stopped. She had a hystero-epileptic convulsion, and left her boarding place and went to the hotel, and without a moment's warning shot him. The jury, after a long and tedious trial, declared her guilty and sentenced her to two years of servitude in the penitentiary.

It was our opinion that she was irresponsible for the killing. The violence of the epileptic paroxysm following his, at least, ungenerous treatment of her, produced a condition of hysterical frenzy that should have relieved her from responsibility, as in the first mentioned case.

Another important question in connection with this matter is the *detection* of hysteria. The tripod of Charcot; the tenderness in the epigastrium ; the tenderness under the left mammary gland; the tenderness over the left dorsal vertebra, and the tenderness over the left ovary; the focal anæsthesia, quoted by Hammond from Chairou ( Diseases of the Nervous System, p. 736). If in a hysterical woman the finger be passed down the throat so as to be brought in contact with the epiglottis, it will be found that this part is quite insensitive, and that it can be rubbed or even scraped with the nail without causing irritation of any kind. Or the superior orifice of the larynx may be similarly treated without exciting either cough or efforts to vomit. Then, again, the peculiar fibrillary movement of the orbicularis palpebrarum when the eyes are closed; the *facies hystericus* with its eye round and full, the upper eyelid drooping, the upper lip remarkably deep and full; the hemi-anæsthesia, especially of the left side; the fact that slight pressure will often produce pain if the attention is fixed, while strong pressure will fail to elicit it if the attention be diverted; the great accumulation of gas in the stomach; the tendency to saliva, and the disorders of nutrition furnish a set of signs that will not often render a diagnosis difficult.

"Alcoholic Trance; its Medico Legal Relations," by T. D. Crothers, Hartford, Connecticut.

"Alcoholic Inebriety," by S. L. Wright, Bellefontaine, Ohio.

### SECTION ON DERMATOLOGY AND SYPHILOGRAPHY.

L. D. Bulkley, New York, Chairman.

F. Dunlap, Danville, Kentucky, Secretary.

Address of the Chairman, "Syphilis as a Non-Venereal Disease."

The discussion on the "Etiology and Treatment of Eczema" was opened by Dr. Bulkley, who, in the course of his remarks, submitted the following table :

- Constitutional and general causes.

  - 1st. Predisposing.

    - Heredity.

    - Gouty or arthritic diathesis.

    - Neurotic diathesis.

    - Strumous diathesis.

  - 2d. Exciting

    - Debility.

    - Digestive disorders.

    - Nervous depression.

- Local or external causes.

  - 1st. Predisposing.

    - Cimatic.

    - Occupation.

      - Sedentary.

      - Standing.

      - Irritating.

    - Diet.

  - 2d. Exciting.

    - Climatic.

      - Sudden exposure to heat and cold.

      - Prolonged exposure.

    - Occupation.

      - Long confinement.

      - Long standing.

      - Irritants.

    - Diet.

      - Acids.

      - Sweets.

      - Tobacco.

    - Irritants.

      - Soaps.

      - Ointments.

      - Scalding.

      - Blister, etc.

Drs. Zeisler and Reynolds of Chicago, Shoemaker of Philadelphia, Ravogli and Rickets of Cincinnati, and Fleisher of New Haven, also took part in the discussion.

"Nervous Causation of Eczema," by E. Chenery, of Boston.

The next paper read was on "A Clinical Study of So-called Prairie Itch, Lumberman's Itch, etc., with a Consideration as to its Entity," by William T. Corlett, M. D., Cleveland, Ohio, in which, in addition to a general résumé of the subject, ten cases were reported, and the opinion was expressed that the disease is really eczema.

Dr. ZEISLER opened the discussion of Dr. Corlett's paper by saying that the cause of the so-called prairie itch was ignorance in regard to skin diseases.

Professor REYNOLDS said that his practice in the West had given him opportunities for seeing many cases of this so-called new disease. He had always found that this diagnosis was made by men who were not especially well posted in dermatology. All the cases of prairie itch he had ever seen were typical cases of eczema. The peculiarity of the prairie itch is the appearance of contagion, which is thought to distinguish it from eczema. This imaginary contagion may be due to the fact that all of a family are under the same hygienic, dietetic, and local conditions. Itching seems to be almost contagious, and the scratching ensuing may occasion a disease of the skin.

The discussion on "The Limit of the Period During which Syphilis can be Communicated by Contagion or Inheritance," was followed by a paper on "The Importance of Local Treatment in Syphilis," by Dr. Joseph Zeisler, of Chicago, Illinois.

Drs. Shoemaker, Dumesnil of St Louis, Palmer, Ravogli, and Keller took part in the discussion.

"Double Chancre *à distance:* An Inquiry into Syphilitic Auto-Inoculation," by A. H. Ohmann Dumesnil, of St. Louis, Missouri.

"Tuberculosis Cutis Verrucosa, with Report of a Case," by George T. Eliot, New York.

Professor Henry J. Reynolds, of Chicago, Illinois, opened this day's session by the reading of a paper on "The Galvanic Method of Treating the Vegetable Parasitic Diseases of the Scalp, with Report of Cases."

Several members participated in the discussion, and the opinion seemed unanimous to the effect that the only influence of the electrical current is the increased penetration of the remedy used in connection with it.

B. M. Ricketts, of Cincinnati, read a paper on "The Use of Arsenic in Dermatology."

He reviewed the history of the use of arsenic in skin diseases, and expressed a preference for recently made solutions of arsenious acid. He uses arsenic in psoriasis and pemphigus.

The opinion developed in the discussion was, upon the whole, not very favorable to a free use of the drug.

Dr. Hale, of Illinois, reported a case of "Purpura Hæmorraghica in a Litttle Girl of Eleven Years."

The eruption first appeared on the abdomen three years ago; it was supposed to be the result of a bruise. Then it spread upon both sides and limbs. Has had slight rheumatic attacks at times. Two months is the longest time she has been free from it. Better now than ever before. She bleeds profusely at intervals of one or two months. There is a history of bleeding in the family for some generations. The aunt and a brother have died from hæmorraghes due to slight causes. There is no history of a specific character.

George T. Eliot, M. D., of New York, read a paper on "Tuberculosis Cutis Verrucosa." The paper was particularly interesting and carefully prepared, but does not admit of sufficient condensation for the purposes of this report.

"On the Value of Frequently-Repeated Doses of Arsenic in the Treatment of Bullous Diseases of the Skin, Especially in Children," by L. Duncan Bulkley, New York.

Arsenic is not so complete a specific as its constant use would seem to indicate. The difficulty is in the failure to adjust the remedy to the particular cases.

He reported a number of cases and said in sufficient doses arsenic is a specific in pemphigus. He prefers a solution of the arseniate of potash or soda.

SECTION ON ORAL AND DENTAL SURGERY.

J. Taft, Cincinnati, Ohio, Chairman.

E. S. Talbot, Chicago, Illinois, Secretary.

Address of Chairman, J. Taft.

"Etiology of Irregularities of the Teeth," "Development of the Superior Maxilla in the Idiot, Deaf and Dumb, and Blind and Insane," by Eugene S. Talbot, Chicago, Illinois.

He explained his views regarding the causes of these malformations:

1. Constitutional causes.
2. Local causes.

He dissents from the views of some authors, who designate them as being congenital.

These deformities, he says, are often so prominent that mechanical interference is necessary to improve the appearance and the use of the teeth.

In his paper he considers the pre-natal and post-natal influence of imperfect nutrition, and consequent imperfect development of the jaw, as influencing the growth of teeth and their position.

Professor J. S. Marshall, of Chicago, in discussing Professor Talbot's paper, said his views regarding congenital origin of such defects did not coincide with the views expressed in Professor Talbot's paper, if he understood the author of the paper as he wished to be understood.

He spoke of the effects of intermarriage between peoples of different nations, and, also, in the same families, showing how improvement may be effected or degeneration result, as is exemplified in some of the inferior animals, and he is inclined to consider these facts and conditions as explaining in part some of these deformities.

A paper on "Fractures and Diastasis of the Superior Maxilla and Upper Bones of the Face, treated by the aid of the Interdental Splint, with two cases as illustrations," by Professor John S. Marshall, of Chicago, Illinois, was read, in which he said this class of injuries has elicited so little attention from the general surgeon or oral specialist that some of the most noted works on surgery make no mention of them. He cited two cases which had been treated by him, and gave the method of treatment of each of them—the history and the result in each case, which was satisfactory.

Professor Talbot, in discussing the paper, said Professor Marshall's paper treats of a subject of great interest, one which demands prompt and positive treatment. Great force is required to produce such injury. Other complications, such as shocks, inflammation, and secondary hæmorrhage are liable to ensue on account of the locality. If nature or treatment can overcome these difficulties another serious one presents itself in devising means to hold the parts in place, after putting them in proper position, until they shall have united.

On one occasion he had been requested to assist in such a case. A girl about eight years of age was struck on the back of the head by a descending elevator, her face being forced to the sill of the building. She sustained a compound fracture of both superior and inferior maxillary bones. The parts were put in their natural position, the teeth being the guide. The jaws were bandaged together in the usual manner. The next morning the bandage was off, and the bones slightly displaced. Where a bandage is used it is not certain that it will remain in position. A skull cap made of cotton cloth to fit the head as far as the eyebrows and occipital protuberance will answer better. A splint for the inferior maxilla may be made of gutta-percha, card-board, tin, or anything that can be molded and fitted to the jaws will do well. Having placed the pieces of bone in position and brought the jaws together, so that the teeth occlude properly, adhesive strips, cut one inch in width, wound and fastened not only to the splint and skull cap but also to the cheeks, will give good support. The strips should be applied in such manner as to exert proper force in the right direction. Three or four should be applied. One or two strips fastened to the splint, the cheeks, and back of the neck will hold all firmly in position.

Such patients can be fed with liquids through a rubber tube. Should the teeth be missing or but a few be in position, occlusion would be impossible and the dental splint must be used. Adhesive strips can then be used in the manner described.

Papers read were upon the "Treatment and Management of the Temporary Teeth," by E. G. Betty, Cincinnati, and "Dentogeny," by W. C. Brittan, Detroit, followed by a paper upon the same subject, by M. H. Fletcher, Cincinnati.

